# Effects of a self-care educational program via telerehabilitation on quality of life and caregiver burden in amyotrophic lateral sclerosis: a single-blinded randomized clinical trial protocol

**DOI:** 10.3389/fpsyg.2023.1164370

**Published:** 2023-08-17

**Authors:** Emília Márcia Gomes de Souza e Silva, Stephano Tomaz da Silva, Ledycnarf Januário de Holanda, Daniel Tezoni Borges, Ana Paula Mendonça Fernandes, Kelly Evangelista Rodrigues da Silva, Tatiana Souza Ribeiro, Luciana Protásio de Melo, Ricardo Alexsandro de Medeiros Valentim, Danilo Alves Pinto Nagem, Ana Raquel Rodrigues Lindquist

**Affiliations:** ^1^Laboratory of Intervention and Analysis of Movement, Department of Physical Therapy, Federal University of Rio Grande do Norte, Natal, Brazil; ^2^Laboratory of Technological Innovation in Health, Federal University of Rio Grande do Norte, Natal, Brazil; ^3^Department of Biomedical Engineering, Federal University of Rio Grande do Norte, Natal, Brazil

**Keywords:** caregivers, amyotrophic lateral sclerosis, telerehabilitation, self-care education, burden, quality of life

## Abstract

**Introduction:**

The implementation of a telerehabilitation protocol for self-care in the routine of caregivers of individuals with amyotrophic lateral sclerosis (ALS) has been associated with reduced levels of stress and improved quality of life. Moreover, it may reduce the difficulty of traveling to perform physical or other self-care activities. Thus, this study designed a clinical trial protocol to investigate the effects of a self-care education program via telerehabilitation on the burden and quality of life of caregivers of individuals with ALS.

**Methods:**

This single-blinded randomized clinical trial will recruit 26 caregivers and randomly allocate them to the experimental (EG = 13) or control group (CG = 13). The EG will receive an informative booklet and participate in a 6-week synchronous telerehabilitation program with a neuropsychologist, nutritionist, and physiotherapist to discuss physical and mental health. The CG will receive an informative booklet on self-care and physical activity and weekly phone calls for 6 weeks to solve questions about the booklet. Outcomes will include the caregiver burden (Zarit scale), quality of life (World Health Organization Quality of Life BREF), pain (McGill Pain Questionnaire), stress (Perceived Stress Scale), and depression (Beck Depression Inventory), which will be evaluated at the baseline after the six-week program and 30 days after the program. Additionally, we will assess daily the nocturnal awakenings, sleep patterns, level of physical activity, and heart rate variability.

**Discussion:**

This study aimed to investigate the effectiveness of telerehabilitation for caregivers of individuals with ALS. If effective, this program could be disseminated among health professionals, increasing the possibility of remotely monitoring individuals with difficulty performing physical activities.

**Trial registration number:**

NCT05884034 (clinicaltrials.gov).

## 1. Introduction

Caregivers have a low quality of life due to the patient worsening, challenges faced with the progression of the health condition, changes in the routine, and reduction in leisure activities (Diniz et al., 2018; Alves et al., 2019; Nogueira et al., 2019; Sanches Slusarski Martins et al., 2022). Although the low quality of life and increased burden of caregivers are well-established characteristics in literature, this topic still requires greater visibility and attention to propose interventions (Burke et al., 2017; Sandstedt et al., 2018).

Informal caregivers (i.e., unpaid individuals who help patients in activities of daily living and medical programming) may also experience issues in family relationships and economic difficulties and suffer from social stigma and physical burden (Ploeg et al., 2017). Approximately 70 to 80% of caregivers are informal; among these, 30 to 68% have anxiety or depression (Wilz and Kalytta, 2008). In addition, the strain increases the risk of cardiovascular diseases and mortality by 23 and 63%, respectively (Richard and Scott, 1999).

Individuals with amyotrophic lateral sclerosis (ALS) present progressive muscle weakness, fatigue, and reduced mobility and functional activities (Lui and Byl, 2009). Moreover, a multidisciplinary team treats ALS focusing on symptoms (Ferreira et al., 2016) since this disease progression cannot be reversed or changed, requiring intermittent care. Informal caregivers are essential to support this population due to the intensive and complex care required (D'Alvano et al., 2022; Poppe et al., 2022). Schischlevskij et al. (2021) found that the functional status and disease severity of individuals with ALS were the main factors influencing caregiver burden. Moreover, cognitive changes (e.g., impaired attention, focus, and memory) and behavioral changes (e.g., irritability, frustration, impatience, and social isolation) in caregivers of individuals with ALS (Burke et al., 2015; Beeldman et al., 2016) and the need to learn how to manage assisted ventilation and enteral feeding has been involved in caregiver burden (Siciliano et al., 2017; Chu and Oh, 2020).

Weight loss in ALS has been associated with reduced survival; thus, healthcare professionals must establish maintaining body weight as a therapeutic goal (Ning et al., 2019; van Mantgem et al., 2020). In this sense, caregivers' knowledge and influence on nutritional behaviors are essential to increase caloric intake and body weight in individuals with ALS (Genton et al., 2011; Marin et al., 2011; Coates et al., 2023). Thus, a support network should be implemented to help with nutritional issues in this disease (Ludolph et al., 2020).

The implementation of physical activity in the routine of caregivers has been associated with reduced stress and improved quality of life and wellbeing (Lambert et al., 2016). However, caregiving demands hamper the adherence to face-to-face programs due to the limited time available, the need for travel, and difficulty finding someone to care for the patient (Winter and Gitlin, 2007). Thus, remote self-care activities are faster and more practical than face-to-face and may be preferred (Hearn et al., 2019) since caregivers can participate in meetings and receive professional guidance and information via video conferences (Van Egmond et al., 2018).

The self-care concept comprises actions to ensure self-health and wellbeing (Lobo et al., 2023). In this sense, caregivers of individuals with ALS need to recognize the importance of self-care since they face great physical and emotional challenges (Young et al., 2023). Moreover, caregivers can maintain their health and quality of life by prioritizing self-care, which improves their ability to care for the patient adequately and efficiently (D'Alvano et al., 2022).

Telerehabilitation is defined as remote rehabilitation via communication technologies that different health professionals can use (e.g., physical, occupational, and speech therapists and psychologists). This tool can be combined with evaluation, monitoring, and health education, may reduce travel costs, and allow flexible schedules and integration into the routine of users (Van Egmond et al., 2018). Moreover, telerehabilitation can be implemented asynchronously (i.e., without real-time contact) or synchronously (i.e., real-time video and audio) using video conferences with one or more individuals simultaneously (Kern, 2006).

Previous studies (Cottrell et al., 2017; Grona et al., 2018; Jiang et al., 2018; Bettger et al., 2020; Prvu Bettger and Resnik, 2020; Yeroushalmi et al., 2020) have shown that telerehabilitation may reduce pain and recover motor function in individuals with musculoskeletal dysfunctions. Moreover, it decreases healthcare costs and improves treatment adherence, physical and mental function, and quality of life (Barlow et al., 2009; Vloothuis et al., 2019).

Educational programs can provide information to cope with the challenges of caregiving and implement relevant topics based on self-care (e.g., sleep health, healthy eating, mental health practices, and physical exercises to support the caregivers' quality of life), allowing informal caregivers to have confidence in providing care and focus on their physical and mental wellbeing (González-Fraile et al., 2021; Zarotti et al., 2021). However, the effects of telerehabilitation on informal caregivers of individuals with ALS are unclear (Watermeyer et al., 2015; Galvin et al., 2018; Burke et al., 2019) since the needs and circumstances may vary for each caregiver and the patient and due to different training programs and telerehabilitation limitations. Moreover, a lack of studies regarding the long-term effects of continuous remote evaluation and telerehabilitation and its efficacy in different disease stages has been observed, hampering to generalize of the studied effects.

In this sense, the following question emerged: Does the quality of life, depression, burden, and stress of caregivers receiving a synchronous telerehabilitation program improve compared with those assisted individually and asynchronously? Thus, we hypothesized that a synchronous telerehabilitation program might reduce the burden, stress, pain, and depression and improve the quality of life of caregivers of individuals with ALS, enhancing their mental and physical health. The benefits may include reduced social isolation and improved access to support and guidance services, coping strategies for stress and emotional burden, and knowledge regarding ALS and adequate care strategies.

This study aimed to design a randomized clinical trial protocol to evaluate the impact of a self-care education program via synchronous telerehabilitation on the quality of life and burden of caregivers of individuals with ALS. All the content addressed in both intervention proposals is innovative, as planned by a multidisciplinary team, aiming to consider different aspects that influence the quality of life, depression, burden, pain, and stress of caregivers. Additionally, the present study will use wearable technologies as a way to monitor and assess physiological states remotely, such as nocturnal awakenings, sleep patterns, level of physical activity, and heart rate variability. Results may show whether remote and synchronous programs favor the self-care of caregivers by improving time and access to health education.

## 2. Methods

### 2.1. Study design

This protocol for a single-blinded randomized clinical trial was developed according to the Standard Protocol Items: Recommendations for Interventional Trials (SPIRIT) (Chan et al., 2013) (see SPIRIT checklist in Supplementary material 1).

### 2.2. Target population and sampling

Participants will comprise informal caregivers of individuals with ALS of both sexes diagnosed by a neurologist (El Escorial criteria) (Brooks et al., 2000), aged 18 years or above, and living in the municipalities of Natal (Rio Grande do Norte, Brazil). They will be recruited using the attendance and waiting list from the Neuromuscular Diseases Outpatient Clinic of the Onofre Lopes University Hospital–HUOL/UFRN/EBSERH. Thus, all participants will be initially contacted through their phone number provided during the registration in these lists.

### 2.3. Power calculation

Sample size was estimated using an online calculator (Dean, 2007) based on caregiver burden (primary outcome), which was evaluated using the Zarit scale. Moreover, the effect size was calculated based on post-training data from Duran Parra et al. (2019) (mean of 16 ± 9.9 and 31.4 ± 14.9 points for experimental and control groups, respectively), with a mean difference of 15.4 between groups. Thus, 22 participants (11 per group) were estimated, and the total sample was determined as 26 participants (13 per group), considering a dropout rate of 20%.

### 2.4. Consent for publication and confidentiality

Information of all participants will be confidential and maintained in a safe place in the laboratory for at least 5 years. Only the corresponding author will access these data, ensuring anonymity, respect, and human dignity. Moreover, the information will only be disclosed anonymously, without initials or indications that could identify the participants.

The findings will be published in peer-reviewed journals and presented at conferences. In case of significant changes in the protocol, we will inform the participants, ClinicalTrials.gov, and journals. A copy of the informed consent form will be provided upon request.

### 2.5. Availability of protocol and data

Information on this randomized clinical trial protocol is registered and available. The corresponding author will provide the study protocol and data supporting the findings upon reasonable request.

### 2.6. Eligibility criteria

#### 2.6.1. Inclusion criteria

- Informal caregiver (i.e., family member, friend, or a caregiver without payment) of an individual with ALS (clinically defined, probable, or possible) diagnosed by a neurologist using the (El Escorial Criteria) (Brooks et al., 2000);
- Informal caregiver of individuals without other neurological diseases associated with ALS (Brooks et al., 2000);
- Aged 18 years or above;
- Without cognitive impairment on the Mini-Mental State Examination (MMSE; cutoff point of 25 for literate individuals) to understand the study and the informed consent form Brucki et al. (2003);
- Literate (at least complete primary education).

#### 2.6.2. Exclusion criteria

- Health conditions hindering exercise safety (e.g., recent surgeries, fractures, uncontrolled heart, vascular, or respiratory disorders, dizziness or vertigo, fainting, oncological diseases, or neurological diseases affecting balance and protective reactions)
- Caregivers in the first trimester of pregnancy (to avoid sudden increase in resting heart rate) (Wolfe and Davies, 2003);
- Using psychiatric medication (e.g., anxiolytics, antidepressants, antipsychotics, or antiepileptics) since they may interfere with the study results
- Absence in the program for two or more consecutive days without replacement.

### 2.7. Randomization, allocation, and blinding

Participants will be randomly allocated into the experimental (EG) or control group (CG). A researcher not involved in the intervention will perform the randomization using the www.randomization.com website and maintain the sequence confidential in opaque sealed and numbered envelopes using codes to identify the groups. The content of envelopes will be revealed to the professionals applying the interventions only at the beginning of the program. The researcher responsible for data analysis will be blinded until the end of the study and will not be involved in interventions or data analysis. Professionals, with the exception of the researcher responsible for data analysis, and participants will not be blinded due to the nature of the intervention.

### 2.8. Intervention groups

#### 2.8.1. Experimental group

A physiotherapist will visit the participants of the EG face-to-face to evaluate their health, deliver the informative booklet, and instruct its use and importance. The booklet titled “Self-care program for caregivers of individuals with ALS” contains guidelines for performing physical activities at home (e.g., stretching, mobility, and strengthening) and information on healthy eating, mental health, and routine management, with a schedule for the weekly organization (Appendix 1).

**Figure 1:**
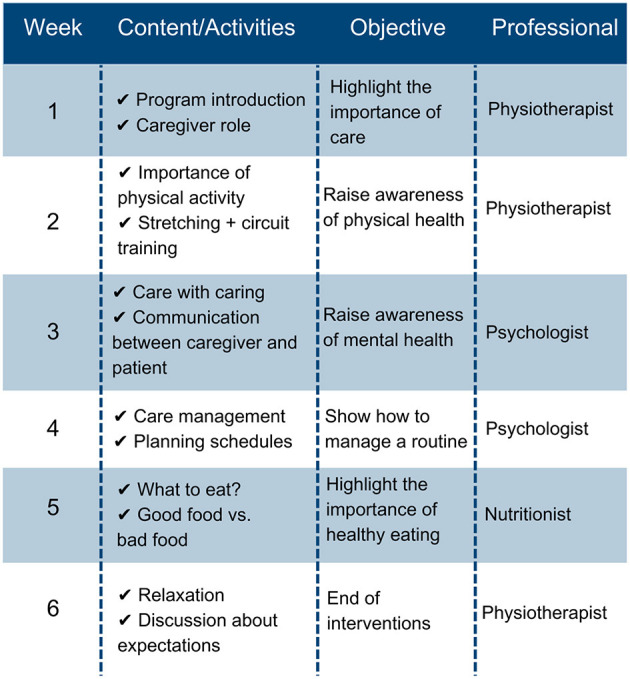
Topics to be addressed in weekly meetings.

Participants in EG will be divided into subgroups (three to five per subgroup). Each subgroup will participate in a 6-week self-care education program with weekly synchronous meetings (Burke et al., 2019; de Wit et al., 2019) using Google Meet or WhatsApp. Each meeting will address topics related to the physical and mental health of caregivers, including the importance of care and caregivers, physical activities to be performed at home, routine management, and healthy eating (Figure 1). A physiotherapist, psychologist, and nutritionist will lead the meetings, which will last approximately 40 min plus a maximum of 20 min to discuss whether the participants' expectations were met.

#### 2.8.2. Control group

**Figure 2:**
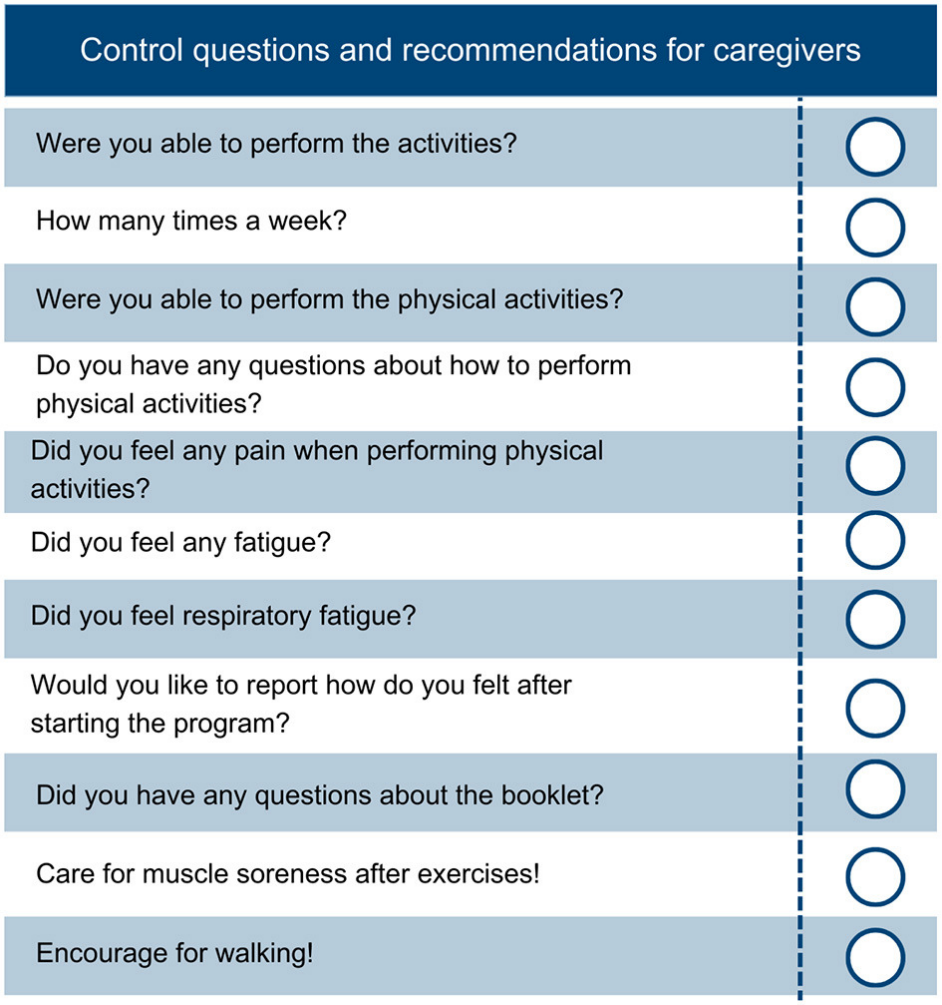
Control questions and recommendations to verify the mental and physical health of participants.

A physiotherapist will also visit the participants in the CG to evaluate their health condition, deliver the informative booklet, and instruct them on the importance of reading and using the booklet (Appendix 1). Next, trained researchers will contact the participants via phone calls during the 2nd to 6th week of the intervention to verify their physical and mental health and collect information on completing physical exercises and habits proposed in the booklet. The researcher should record any relevant information. Figure 2 shows the questions for participants. After the intervention period, a new visit will be performed for revaluation.

### 2.9. Study procedures

**Figure 3:**
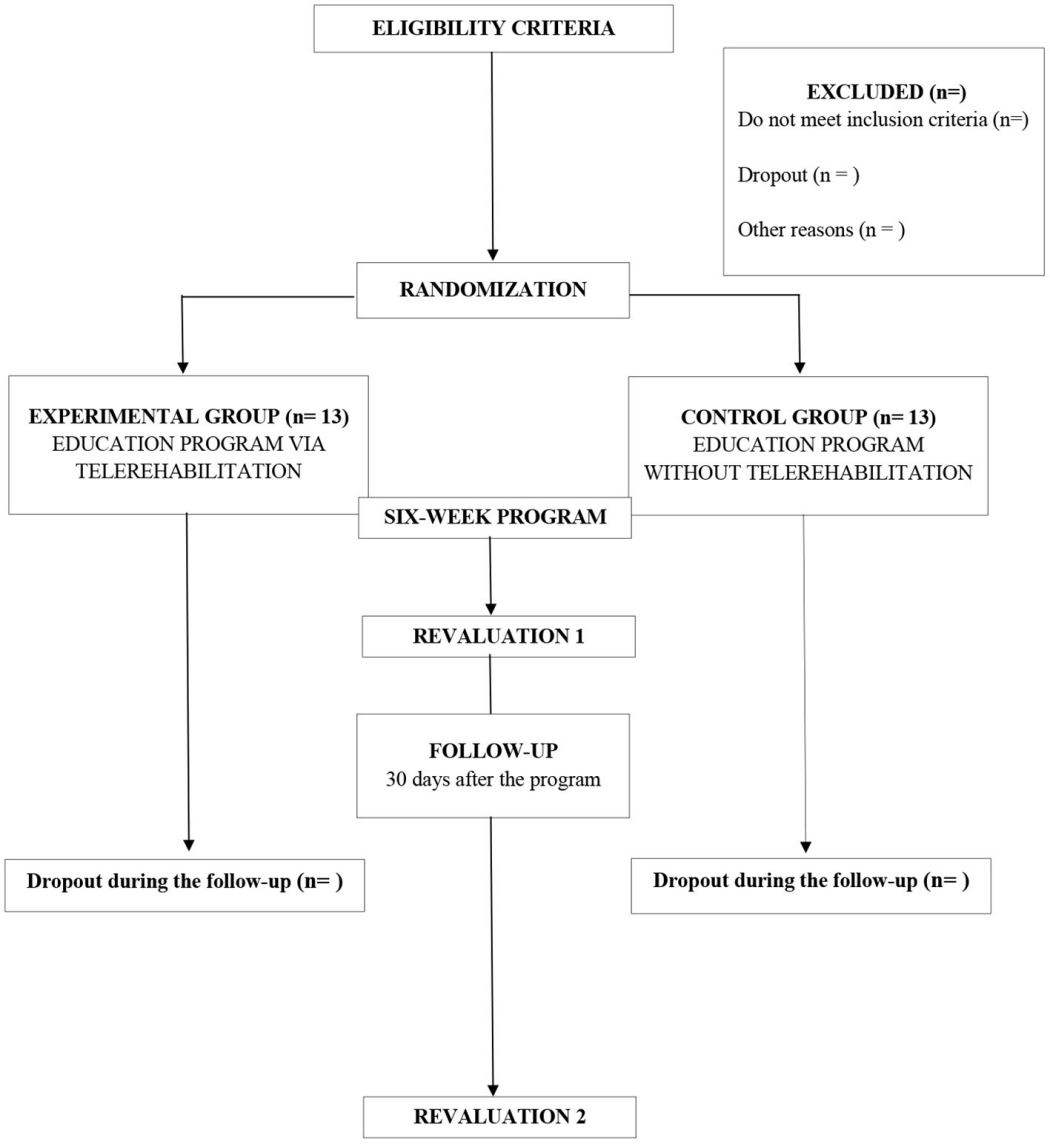
Flowchart of the study.

After receiving the informative booklet, participants will respond to questionnaires to characterize the sample and evaluate cognitive function. In the baseline evaluation, the caregiver burden, quality of life, pain, stress, and depression will be evaluated. Next, participants will be randomly assigned to the CG or EG. Outcomes will be revaluated after the 6-week program (revaluation 1) and 30 days after the program (revaluation 2) (Figure 3). If the EG shows better results than CG at the end of the study, the same intervention will be available for CG to ensure equality of treatment for all participants.

### 2.10. Sample characteristics

#### 2.10.1. Sociodemographic, clinical, and anthropometric evaluation

During the delivery of the informative booklet, anthropometric data (i.e., height and weight) will be collected using a measuring tape and portable digital scale. Sex, time spent caring for a patient (in months), time of ALS diagnosis, relationship of the individual with ALS, profession, and age will also be collected. These data will be recorded on the identification form to characterize the sample.

#### 2.10.2. Physical evaluation and time spent caring for a patient

Participants will be asked about physical activity type and frequency. Moreover, data regarding how long (in months) the participants have been caring for a patient (with any condition) and whether the participant performs the care for the same individual will be collected.

#### 2.10.3. Cognitive function

Cognitive function will be evaluated using the MMSE (Brucki et al., 2003), which evaluates orientation to time and place, short-term memory (recall), attention, language skills, visuospatial abilities, and ability to understand and follow instructions. The total score ranges from 0 to 30; high scores indicate greater cognitive function. In addition, the cutoff point of 25 will be used for literate individuals (Brucki et al., 2003).

### 2.11. Outcomes

Outcomes will be evaluated at the baseline, immediately after the 6-week program, and 30 days after the end of the program. A researcher not involved in the intervention will remotely reassess all participants via *Whatsapp* or *Google Meet*.

#### 2.11.1. Primary outcomes

- Caregiver burden

Considering that studies have indicated a high level of burden in caregivers of individuals with ALS (de Wit et al., 2018), this outcome will be evaluated using the Zarit scale, a widely used instrument in both research and clinical practice, to assess the level of burden and stress experienced by caregivers of people with disabilities. This scale enables a quantitative assessment of perceived burden, facilitating the identification of caregivers at risk of chronic stress and exhaustion. Furthermore, it aids in planning appropriate interventions and support services for caregivers. This scale has been widely used and has an intraclass reliability coefficient of 0.88 and Cronbach's coefficient alpha of 0.77 (Taub et al., 2004). It consists of 22 questions that are scored on a 5-point Likert scale, ranging from 0 (never) to 4 (nearly always) (Al-Rawashdeh et al., 2016). The maximum score is 88 points; high scores indicate a greater level of caregiver burden (Taub et al., 2004). Moreover, the Zarit scale specifically addresses the caregiver-patient relationship and evaluates various aspects of the caregiver's life, such as physical and mental health (“Do you feel you do not have time for yourself because of the person's care?”), economic status (“Do you feel that the person's care has affected your financial wellness?”), and social wellbeing (“Do you think that your personal life was affected owing to the person's care?”).

- Quality of life

The quality of life will be evaluated using the short version of the World Health Organization Quality of Life-BREF (WHOQOL-BREF) (Orley et al., 1995), which consists of 26 questions and has a Cronbach's coefficient alpha of 0.91. The first question refers to the general quality of life, and the second to satisfaction with health. The remaining 24 questions are divided into four domains: physical (e.g., “How satisfied are you with your health?”), psychological (e.g., “How often do you have negative feelings such as blue mood, despair, anxiety, depression?”), and environmental health and social relationships (e.g., “How satisfied are you with the support you receive from your friends?”). This scale specifically focuses on evaluating the caregiver-patient relationship and the physical and mental health, economic status, and social wellbeing of caregivers (Fleck et al., 2000; Vahedi, 2010) since caregivers of individuals with ALS experience a significant burden (Pagnini et al., 2010).

#### 2.11.2. Secondary outcomes

- Pain

Several studies have reported that informal caregivers have an increased risk of experiencing pain, such as musculoskeletal pain and headaches (Darragh et al., 2015; Llamas-Ramos et al., 2023). Thus, the pain will be evaluated using the McGill Pain Questionnaire, a well-established tool with reasonable validity and reliability (Pimenta and Teixeira, 1996) and a Cronbach's coefficient alpha of 0.96 (Lovejoy et al., 2012). This questionnaire consists of 78 descriptors of pain, which are organized into four categories (sensory, affective, evaluative, and miscellaneous) and divided into 20 subcategories. Participants will be asked to carefully select a word from each subcategory that best represents their personal experience of pain, with an option to abstain from selecting any word if needed, e.g., in the first subcategory, the evaluated person will have the following word options: (1) vibration, (2) tremor, (3) pulsating, (4) throbbing, (5) knocking, (6) bumping (Pimenta and Teixeira, 1996).

- Stress

Considering that caregiving may increase the psychological and physiological stress of the caregiver (Penning and Wu, 2016; Bidwell et al., 2021; Tough et al., 2022), stress will be evaluated using the Perceived Stress Scale (Luft et al., 2007). This scale has demonstrated good reliability and validity and a Cronbach's coefficient alpha of 0.82 (Luft et al., 2007). It comprises 14 questions, with responses scored from 0 (never) to 4 (very often), e.g., “Are you feeling nervous and stressed?”. The total score is obtained by summing all individual scores (ranging from 0 to 56); high scores indicate a greater level of perceived stress (Luft et al., 2007).

- Depression

Depressive symptoms will be evaluated using the Beck Depression Inventory (Beck et al., 1961) since the caregiver burden may cause mental changes and increase the risk of depression over the years of caregiving (Gauthier et al., 2007). This widely used tool has demonstrated good reliability and validity and a Cronbach's coefficient alpha of 0.9 (Toledano-Toledano and Contreras-Valdez, 2018). It consists of 21 items evaluating various attitudes and symptoms related to depression [e.g., sadness (“I feel sad or discouraged?”), pessimism (“I feel hopeless about the future”), loss of pleasure, guilt, self-criticism, suicidal thoughts, changes in sleeping and eating patterns, fatigue, and decreased libido]. Each item is scored on a scale of 0 (indicating the absence or minimal presence of the symptom) to 3 (representing severe presence of the symptom), with higher scores indicating a higher level of depression. (Beck et al., 1961).

- Nocturnal awakenings

Nocturnal awakenings will be evaluated using the *Sênior Saúde Móvel* platform (Rodrigues et al., 2022), a new wearable device to monitor outcomes related to health, such as nocturia. Nocturia is the need to wake up during sleep to urinate, with each urination preceded and followed by sleep (International Continence Society, 2018). This condition causes significant discomfort and has shown a high prevalence in adults (Vaughan and Bliwise, 2018).

- Sleep patterns

Sleep patterns will also be evaluated using the *Sênior Saúde Móvel* platform (Rodrigues et al., 2022). Studies have shown that sleep disturbances are closely related to increased caregiver burden (Leggett et al., 2018; Perez et al., 2022). Moreover, hypervigilance, cohabitation with the individual being cared for, and caregiver tension have been associated with sleep impairments in caregivers. Thus, this platform will allow us to collect comprehensive data on the sleep quality and patterns of caregivers, providing valuable insights into their overall wellbeing.

- Level of physical activity

The level of physical activity will be analyzed using the *Sênior Saúde Móvel* platform (Rodrigues et al., 2022) since it predicts physical and psychological stress which can impact caregivers' health (Stults-Kolehmainen and Sinha, 2014).

- Heart rate variability

Considering that stress, burden, sleep disturbances, and fatigue may change the cardiovascular system, especially the heart rate, its variability can be used to screen caregivers' health. Van Der Zwan et al. (2015). Thus, the heart rate variability will be analyzed using the *Sênior Saúde Móvel* platform (Rodrigues et al., 2022).

### 2.12. Adverse events

Adverse events will be self-reported and recorded during the weekly contact with the participant during the intervention (via video or phone call), or at other periods (via message and informative booklet). However, if participants need to report any occurrence, they can contact the researchers at any moment. Potential adverse events will guide adaptations to the exercise program and help the researchers to manage difficulties faced by the caregivers.

### 2.13. Adherence rate

Adherence to the intervention will be calculated by the percentage of participants who completed the 6-week program. Moreover, adherence strategies (e.g., phone calls) will remind participants of sessions and evaluations, and schedules will be flexible and adjusted according to the needs of participants. Potential issues hampering participation or continuity in the study will be prevented or solved, such as unexplained absence in the interventions, conflicting schedules with the activities of caregivers (e.g., meals and hygiene of the individual with ALS or caregiver), and inadequate environments (e.g., noisy or lacking privacy). These issues will be solved by guiding the caregivers to perform the intervention in a calm environment (i.e., without distractions) and adjust their daily routine to spend time on health improvement and rescheduling the session days or times if needed. Two absences without replacement, illness, disorder, or persistent pain hampering continuity will be considered non-adherence to the program.

### 2.14. Statistical analysis

Data will be analyzed using the Statistical Package for the Social Sciences software version 22.0 (IBM Corp., Chicago, IL, USA). Data will be initially characterized using descriptive analysis, and the significance level will be set at 5%. The Shapiro-Wilk test will be applied to verify data normality. Differences between groups on different evaluation periods will be analyzed using linear mixed models. The Bonferroni *post hoc* may be used for statistically significant results to identify specific differences. Moreover, an intention-to-treat analysis will be used to compare the groups by including all participants as allocated after randomization, ensuring an unbiased approach. Participants' data will be analyzed based on their intervention group, regardless of any dropouts. Values from the previous evaluation will be replicated and attributed to missing data due to dropout.

## 3. Discussion

This is the first study comparing a synchronous (follow-up via video call) and asynchronous (follow-up via messages and informative booklet) multidisciplinary self-care educational program for caregivers of individuals with ALS. According to Chi and Demiris (2015), caregivers approve and feel comfortable using telerehabilitation to improve self-care and quality of life. In this sense, telerehabilitation has been growing in the healthcare field and can be a useful tool to promote physical activities and self-care.

Despite being aware of the benefits of physical activity, caregivers face barriers, such as lack of support from family members, self-motivation, and time to perform self-care activities, guilty feelings, and stress (Farran et al., 2008; Marquez et al., 2012; Martin and Keats, 2014). Most caregivers have reported difficulty performing self-care activities because they prioritize patient care (Son et al., 2007; Farran et al., 2008; Snyder and Vitaliano, 2020). In this sense, these barriers will be addressed in this multidisciplinary study, highlighting the need for interventions involving different professionals (e.g., psychologists, and nutritionists) for the biopsychosocial management of caregivers. Telerehabilitation aims to facilitate adherence to this program.

In this study, all participants will receive the informative booklet on health and self-care from a multidisciplinary team, allowing easy access to this information. This study aims to understand whether monitoring and providing synchronous information result in better outcomes than asynchronous information. It may contribute to the knowledge about the applicability of telerehabilitation for caregivers of individuals with ALS and reinforce the need for self-care.

This study has limitations, such as the possible sociodemographic homogeneity of caregivers, considering that they will be recruited in the same region. Caregivers will read the informative booklet and perform the physical activity individually and spontaneously throughout the days of the week due to their routines. Although this self-applied intervention may present low reliability, the *Sênior Saúde Móvel* platform will allow greater accuracy of the level of physical activity. The exercise guidelines are not based on the specific needs and characteristics of caregivers and may not have good applicability for all; however, caregivers will be asked to report on possible limitations and difficulties in performing some exercises, which may be adapted through professional guidance. In addition, the EG will receive the interventions via group video calls. In this sense, the follow-up of the groups might be difficult throughout the weeks due to different caregivers' demands, hampering the evaluation of the effect of the intervention in groups.

To solve the mentioned limitations, caregivers will receive detailed information regarding the booklet and be able to solve any doubt at the first evaluation. A team will be available to support the caregivers in performing the exercises and applying the booklet in their daily routines during the program via WhatsApp groups. The *Senior Saúde Móvel* platform will be used for remote data collection to ensure reliability in evaluating the proposed program, allowing identification and monitor the adherence and execution of the program.

## Ethics statement

This study was approved by the research ethics committee of the Federal University of Rio Grande do Norte (no. 4.076.825/20) and registered in the ClinicalTrials.gov (NCT05884034). All participants will be informed about the aim of the study, highlighting the importance of self-care and strategies to implement the practices discussed in the booklet in their daily living, before the program and sign the informed consent form, following the Resolution 466/2012 of the National Health Council and the Declaration of Helsinki.

## Author contributions

EG designed the clinical trial and wrote this manuscript. ST, LJ, AM, and KE wrote the protocol. LP and TS supported developing the intervention protocol and wrote this manuscript. RdM and DA reviewed and criticized the manuscript. AR organized the research project and reviewed and criticized the manuscript. All authors substantially contributed, read, and approved the final version of this protocol.
