# Supplementary material for: Effects of a self-care educational program via telerehabilitation on quality of life and caregiver burden in amyotrophic lateral sclerosis: a single-blinded randomized clinical trial protocol
Source: Front Psychol. 2023 Aug 17;14:1164370. doi: 10.3389/fpsyg.2023.1164370 (PMC10472276; doi:10.3389/fpsyg.2023.1164370)
Supplement: Supplementary file 1 [file Data_Sheet_1.PDF]

Additional File 1: Study Schedule (SPIRIT diagram)

	STUDY PERIOD					
	**Enrolment**	**Allocation**	**Post-allocation**			**Post treatment**
**TIMEPOINT****	**0**	**0**	**1-2**	**3-4**	**5-6**	**4 weeks after treatment**
**ENROLMENT:**						
**Eligibility screening**	X					
**Informed consent**	X					
**Allocation**		X				
**INTERVENTIONS:**						
Experimental group			◆—	—	—◆	
Control group			◆—	—	—◆	
**EVALUATIONS:**						
Caregiver burden/Zarit scale	X				X	X
Quality of life/WHOQOL-BREF	X				X	X
Pain/McGill	X				X	X
Stress/PSS	X				X	X
Depression/BDI	X				X	X
Sociodemographic data	X					
Night awakenings	X				X	X
Sleep patterns	X				X	X
Levels of physical activity	X				X	X
Heart rate variability	X				X	X

WHOQOL-BREF: short version of the World Health Organization Quality of Life-100; McGill: McGill Pain Questionnaire; PSS: Perceived Stress Scale; BDI: Beck Depression Inventory.
